# A hybrid 2D/4D‐MRI methodology using simultaneous multislice imaging for radiotherapy guidance

**DOI:** 10.1002/mp.15802

**Published:** 2022-06-22

**Authors:** Katrinus Keijnemans, Pim T. S. Borman, Prescilla Uijtewaal, Peter L. Woodhead, Bas W. Raaymakers, Martin F. Fast

**Affiliations:** ^1^ Department of Radiotherapy University Medical Center Utrecht Heidelberglaan 100 3584 CX Utrecht The Netherlands; ^2^ Elekta AB kungstensgatan 18 113 57 Stockholm Sweden

**Keywords:** 4D‐MRI, lung cancer, MLC tracking, MR‐linac, simultaneous multislice

## Abstract

**Purpose:**

Respiratory motion management is important in abdominothoracic radiotherapy. Fast imaging of the tumor can facilitate multileaf collimator (MLC) tracking that allows for smaller treatment margins, while repeatedly imaging the full field‐of‐view is necessary for 4D dose accumulation. This study introduces a hybrid 2D/4D‐MRI methodology that can be used for simultaneous MLC tracking and dose accumulation on a 1.5 T Unity MR‐linac (Elekta AB, Stockholm, Sweden).

**Methods:**

We developed a hybrid 2D/4D‐MRI methodology that uses a simultaneous multislice (SMS) accelerated MRI sequence, which acquires two coronal slices simultaneously and repeatedly cycles through slice positions over the image volume. As a result, the fast 2D imaging can be used prospectively for MLC tracking and the SMS slices can be sorted retrospectively into respiratory‐correlated 4D‐MRIs for dose accumulation. Data were acquired in five healthy volunteers with an SMS‐bTFE and SMS‐TSE MRI sequence. For each sequence, a prebeam dataset and a beam‐on dataset were acquired simulating the two phases of MR‐linac treatments. Prebeam data were used to generate a 4D‐based motion model and a reference mid‐position volume, while beam‐on data were used for real‐time motion extraction and reconstruction of beam‐on 4D‐MRIs. In addition, an in‐silico computational phantom was used for validation of the hybrid 2D/4D‐MRI methodology. MLC tracking experiments were performed with the developed methodology, for which real‐time SMS data reconstruction was enabled on the scanner. A 15‐beam 8× 7.5 Gy intensity‐modulated radiotherapy plan for lung stereotactic body radiotherapy with isotropic 3 mm GTV‐to‐PTV margins was created. Dosimetry experiments were performed using a 4D motion phantom. The latency between target motion and updating the radiation beam was determined and compensated. Local gamma analyses were performed to quantify dose differences compared to a static reference delivery, and dose area histograms (DAHs) were used to quantify the GTV and PTV coverage.

**Results:**

In‐vivo data acquisition and MLC tracking experiments were successfully performed with the developed hybrid 2D/4D‐MRI methodology. Real‐time liver–lung interface motion estimation had a Pearson's correlation of 0.996 (in‐vivo) and 0.998 (in‐silico). A median (5th–95th percentile) error of 0.0 (−0.9 to 0.7) mm and 0.0 (−0.2 to 0.2) mm was found for real‐time motion estimation for in‐vivo and in‐silico, respectively. Target motion prediction beyond the liver–lung interface had a median root mean square error of 1.6 mm (in‐vivo) and 0.5 mm (in‐silico). Beam‐on 4D MRI reconstruction required a median amount of data equal to an acquisition time of 2:21–3:17 min, which was 20% less data compared to the prebeam‐derived 4D‐MRI. System latency was reduced from 501 ± 12 ms to −1 ± 3 ms (SMS‐TSE) and from 398 ± 10 ms to −10 ± 4 ms (SMS‐bTFE) by a linear regression prediction filter. The local gamma analysis agreed within −3.8% to 3.3% (SMS‐bTFE) and −5.3% to 10% (SMS‐TSE) with a reference MRI sequence. The DAHs revealed a relative $D_{98\%}$ GTV coverage between 97% and 100% (SMS‐bTFE) and 100% and 101% (SMS‐TSE) compared to the static reference.

**Conclusions:**

The presented 2D/4D‐MRI methodology demonstrated the potential for accurately extracting real‐time motion for MLC tracking in abdominothoracic radiotherapy, while simultaneously reconstructing contiguous respiratory‐correlated 4D‐MRIs for dose accumulation.

## 1 INTRODUCTION

Motion management in the abdominal and thoracic regions is important for external beam radiotherapy.^1^, ^2^, ^3^ Respiratory and gastrointestinal‐induced motion causes movements in patient anatomy that are typically of the order of 1–3 cm.^4^, ^5^, ^6^, ^7^ Respiration contributes most to tumor motion, and, therefore, large treatment margins are used in stereotactic body radiotherapy (SBRT) to prevent underdosage of the tumor.^1^, ^8^

Magnetic resonance imaging (MRI) is well suited for motion estimation due to its superior soft tissue contrast and acquisition flexibility.^9^ With the introduction of integrated MR linac (MR‐linac) systems,^10^, ^11^ online MRI‐guided radiotherapy workflows were enabled that can estimate motion during treatment.^3^, ^12^ The MRIdian MR‐linac (ViewRay Inc., Oakwood Village, OH) features a 0.35 T MRI scanner,^13^ while the Unity MR‐linac (Elekta AB, Stockholm, Sweden) features a 1.5 T MRI scanner.^14^ For MR‐guided radiotherapy on the MR‐linacs, it is desirable to (1) have fast imaging of the target for real‐time motion management and (2) simultaneously perform full field‐of‐view (FOV) imaging for 4D dose accumulation.^9^ The first condition facilitates beam gating and multileaf collimator (MLC) tracking, which reduces the treatment margins and thereby dose to the organs at risk.^15^, ^16^ Although beam gating prolongs treatment times, MLC tracking maintains a 100% duty cycle.^15^, ^17^ The second condition enables intrafraction anatomical variations to be captured during treatment through respiratory‐correlated 4D‐MRIs. As a result, delivered dose can be more accurately quantified.^18^

Fulfilling both requirements simultaneously is challenging because full FOV (3D) imaging does not yet meet the temporal necessity needed in abdominothoracic radiotherapy.^19^ To overcome this limitation, various techniques combining fast (2D) imaging and dose accumulation have been developed over the years.^20^ Menten et al. utilized 2D imaging in combination with treatment log files for dose accumulation in prostate radiotherapy. Only translational motion was extracted from sagittal 2D cine‐MR images with an update frequency of 1.63 Hz, which is insufficient for abdominothoracic radiotherapy, and applied to a prebeam acquired 3D MRI volume. For abdominothoracic radiotherapy purposes,^21^ Stemkens et al. developed a 4D‐MRI method in which synthetic beam‐on 4D‐MRIs were created by applying a prebeam‐derived motion model (using principal component analysis) to real‐time acquired 2D cine‐MR images. In a similar fashion, a phantom study was performed by Rabe et al.,^22^ in which 4D‐MRIs were estimated using a propagation method on the MRIdian MR‐linac.^23^ To progress toward nonsynthetic image volumes for dose accumulation,^24^ Paulson et al. developed a 3D‐based golden‐angle radial stack‐of‐stars (GAR SoS) 4D‐MRI workflow for the Unity MR‐linac. However, only prebeam and postbeam 4D‐MRIs were reconstructed and used for dose accumulation instead of beam‐on 4D‐MRIs.^25^ Liu et al. showed the potential of using 3D GAR SoS for MRI‐based motion estimation. A limitation, however, is that motion must be estimated from undersampled radial k‐space samples in combination with a prebeam‐ derived motion model.^26^ Mickevicius et al. were the first to show the combination of fast 2D imaging of the target and simultaneously acquiring slices to image the volume. However, their technique is limited to sequences using gradient spoiling and has saturation bands in the images as orthogonal slices are acquired.

This study aims to demonstrate a novel hybrid 2D/4D‐MRI methodology facilitating fast 2D imaging and simultaneously obtaining respiratory‐correlated 4D‐MRIs suitable for abdominothoracic radiotherapy. The proposed methodology is based on the previously developed simultaneous multislice (SMS) accelerated 4D‐MRI sequence, which has the flexibility of choosing the desired contrast.^27^ First, we developed the hybrid 2D/4D‐MRI methodology consisting of a prebeam and beam‐on phase. In the prebeam phase, a reference volume and 4D‐based motion model are built, which are then used in the beam‐on phase for real‐time motion estimation. An in‐silico computational phantom was used for validation of the proposed methodology. Second, we performed MLC tracking experiments using the hybrid 2D/4D‐imaging. We enabled real‐time data reconstruction to avoid latency during SMS reconstruction, and compared the dosimetric results of the tracking experiments with 2D cine‐MRI tracking.

## 2 MATERIALS AND METHODS

### 2.1 Overview hybrid 2D/4D‐MRI workflow

**FIGURE 1:**
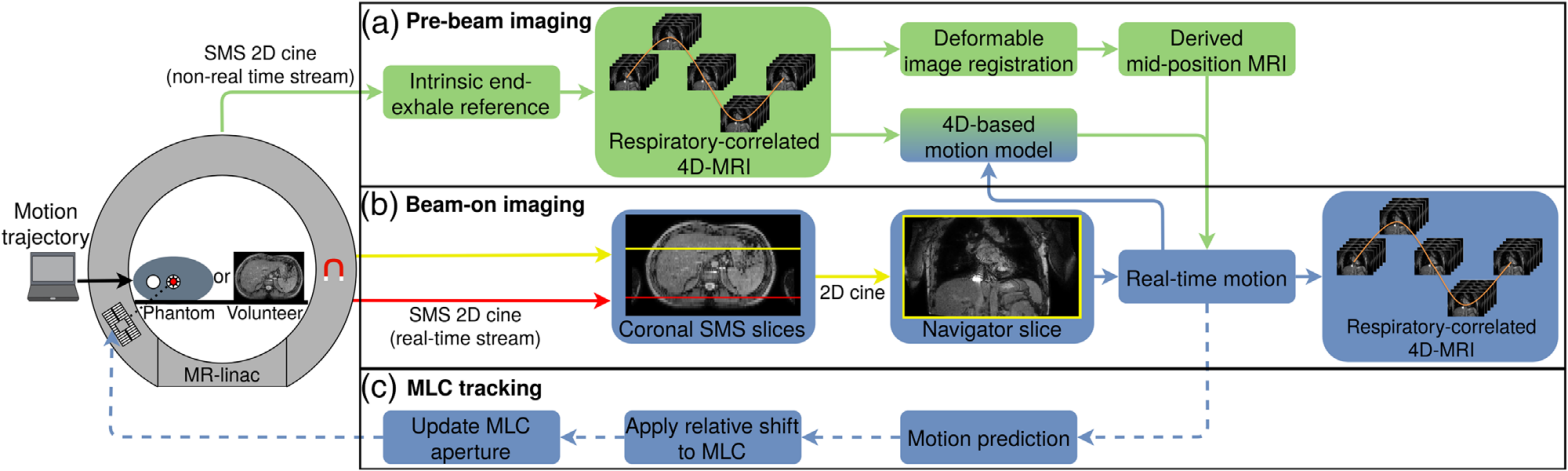
The novel hybrid 2D/4D‐MRI methodology. (a) Simultaneous multislice (SMS) images are used to create a reference mid‐position volume and 4D‐based motion model in the prebeam imaging phase. (b) Continuous SMS acquisition estimating real‐time motion by registering the navigator slice to the mid‐position and applying the 4D‐based motion model. The motion model is continuously updated and contiguous beam‐on 4D‐MRIs are reconstructed. (c) Multileaf collimator (MLC) tracking based on estimated real‐time motion

The proposed hybrid 2D/4D‐MRI methodology is depicted in Figure 1. It consists of three parts during an MR‐linac treatment: (a) represents the prebeam imaging phase, (b) the beam‐on imaging phase, and (c) represents MLC tracking based on the beam‐on imaging during treatment. In the prebeam imaging phase, SMS data are acquired and retrospectively sorted into a respiratory‐correlated 4D‐MRI using an intrinsic end‐exhale reference. Subsequently, a mid‐position volume is derived from the sorted 4D‐MRI using deformable image registration. In addition, the sorting process provides a 4D‐based motion model. During the beam‐on imaging phase, the 4D‐based motion model and mid‐position volume are used to extract real‐time motion from the acquired SMS data, and the SMS data are sorted into beam‐on 4D‐MRIs. The MLC tracking component forms a closed loop with the beam‐on imaging phase, where the continuous SMS data stream provides the target location for MLC tracking. The latency that is involved as a result of image‐based target motion estimation and MLC adjustment is compensated for by a predictor. Section 2.2 will describe the two MR‐linac imaging phases in more detail and Section 2.3 will describe the MLC tracking experiments that were performed.

All (in‐vivo) MRI acquisitions and MLC tracking experiments were performed on a 1.5 T Unity MR‐linac (Elekta AB) in research mode. For validation of the real‐time motion estimation, we simulated MRI acquisitions with the 4D extended cardiac‐torso (XCAT) phantom.^28^ The Quasar MRI^4*D*^ phantom (Modus Medical Devices Inc., London, ON, Canada) was used during all MLC tracking experiments.

### 2.2 Prebeam and beam‐on imaging

#### 2.2.1 Data acquisition

##### In‐vivo

MRI examinations were performed in five healthy volunteers who gave informed consent. Data acquisition was carried out using our previously developed SMS accelerated 4D‐MRI sequence.^27^ This sequence acquires two coronal slices simultaneously (separated by half the FOV in anterior‐posterior [AP] direction) and repeatedly cycles through slice positions over the image volume. Data were acquired with an SMS accelerated single‐shot *T*_2_/*T*_1_‐weighted balanced turbo field echo (bTFE) and *T*_2_‐weighted turbo spin echo (TSE) sequence covering a stack of 52 slices with each dynamic (350(CC)× 457(LR)× 208–260(AP) mm^3^ FOV). Per sequence type, two SMS scans were acquired: a prebeam scan (30 dynamics) and a beam‐on scan (90 dynamics) simulating the two phases of MR‐linac treatments. A beam‐on scan of 90 dynamics was chosen to be able to reconstruct multiple beam‐on 4D‐MRIs. The prebeam scans took 5:35 and 4:18 min, respectively, for the SMS‐bTFE and SMS‐TSE sequences, and the beam‐on scans took 16:32 and 12:24 min, respectively.

To maximize comfort during beam‐on imaging, specific absorption rate and the maximum slew rate were reduced to limit heat deposition and acoustic noise, respectively. The ensuing increase in dynamic scan time was compensated by decreasing the in‐plane acquisition resolution from 2(CC)× 2(LR) mm^2^ to 2× 2.5 mm^2^, while keeping the same reconstructed in‐plane resolution (1.9× 1.9 mm^2^).

For motion estimation validation, a 4 Hz coronal 2D‐bTFE sequence was acquired for approximately 1 min (350(CC)× 457(LR)× 10(AP) mm^3^ FOV, 2× 2.5 mm^2^ in‐plane resolution). Further scan parameters of the in‐vivo acquired SMS and 2D‐bTFE sequences can be found in the Supplementary Materials (Table S1).

##### In‐silico

The in‐plane resolution (2× 2 mm^2^), matrix size (240× 240), slice thickness (5 mm), and number of slices (52) of the XCAT phantom were matched to actual acquired SMS scans. In addition, a spherical lesion of 20 mm in diameter was simulated in the right lung of the XCAT phantom. A series of 3D volumes was generated with both cardiac and respiratory motions. The cardiac period was set to 1 s, and the cardiac motion due to breathing was 5 mm in AP and 20 mm in CC direction. A cos^4^ respiratory trajectory with amplitude and period variation was implemented, having a peak‐to‐peak amplitude between 23 and 28 mm and a respiratory period between 4.4 and 5.6 s. Four breathing scenarios were simulated: one trajectory without drift, two trajectories with cranial drift (0.5 and 1 mm/min) of liver and tumor, and one trajectory with cranial drift (0.5 mm/min) of the liver only. Per scenario, 3120 volumetric datasets were created to simulate a prebeam (30 dynamics) and beam‐on (90 dynamics) scan. From the created 3D volumes, SMS slices were extracted in an interleaved order simulating an actual SMS acquisition.

#### 2.2.2 Processing of prebeam and beam‐on images

##### Prebeam imaging data

The prebeam data were used to create an end‐exhale reference volume by registering (normalized cross‐correlation) the liver–lung interface off all dynamics to each other in each navigator slice location. The navigator slice is the slice of the SMS pair that captures the liver–lung interface, and cycles with the interleaved SMS acquisition. Then, the navigator slices were registered (normalized cross‐correlation) to this intrinsic end‐exhale reference volume to obtain relative cranial‐caudal (CC)‐motion. Nonuniform CC‐motion across AP slice locations in the liver was accounted for by using a 4D‐based motion model, which scales the CC‐motion per slice location. Sorting the scaled CC‐motion in temporal acquisition order results in a unified self‐sorting signal representing liver dome CC‐motion. A sorted 4D‐MRI was created by applying amplitude binning on the self‐sorting signal.^27^ No gradient nonlinearity (GNL) correction was performed, as no 3D correction is available for 2D acquisitions on the scanner.^29^ A mid‐position volume was derived from the sorted SMS‐4D‐MRI using deformable image registration,^30^ which has less noise and artifacts compared to the individual 4D‐MRI phases.^27^ The 4D‐based motion model and mid‐position volume served as reference for beam‐on imaging.

##### 4D‐based motion model

The 4D‐based motion model is necessary to obtain a unified self‐sorting signal for sorting of the prebeam data, which we validated in our previous work.^27^ To this end, the standard deviation of the CC‐motion relative to the end‐exhale reference volume was determined and used for motion amplitude normalization. Similarly, the 4D‐based motion model is required to obtain a real‐time estimate of liver–lung interface motion for the coronal slice intersecting the treatment target during beam‐on imaging. This is necessary because the treatment target is not captured with every single navigator slice of the interleaved SMS acquisition pattern. Therefore, the prebeam‐derived 4D‐based motion model was used to extract real‐time motion from the beam‐on images at the start and then continuously updated based on the most recent 30 dynamics before use (sliding window). This updating took place after processing each subsequent dynamic of beam‐on data, by re‐evaluating the standard deviation of the CC‐motion in each navigator slice location of the most recent 30 dynamics of data.

##### Beam‐on imaging data

The beam‐on data were used for real‐time motion estimation by rigid registration (of the navigator slice) relative to the prebeam mid‐position image and applying the prebeam and updated 4D‐based motion model. In addition, the beam‐on data were used to create contiguous 4D‐MRIs while minimizing the acquisition time per beam‐on 4D‐MRI. To this end, a dynamic inclusion criterion resulting in 10% of missing data in the 4D‐MRI was deployed and offline 3D GNL correction was applied to ensure physiological plausible 4D‐MRIs.^27^, ^31^ An extra step was taken during processing of the beam‐on SMS‐bTFE data. These data had a lower signal‐to‐noise ratio compared to other datasets, and for some volunteers, off‐resonance effects were visible in the region of interest for the rigid registration. The images were binarized using two‐thirds of Otsu's threshold, which was found to binarize the liver–lung interface correctly by visual inspection.

#### 2.2.3 Correlation model

To facilitate real‐time motion estimation for treatment targets beyond the liver dome, a linear correlation model was developed that estimates treatment target motion based on liver–lung interface motion. For the healthy volunteers, a correlation model between liver–lung interface motion and spleen–lung interface motion was built using template matching (normalized cross‐correlation) in a region of interest at the corresponding interfaces in the prebeam images. This model was then applied to the liver–lung interface motion in the beam‐on images to predict the spleen–lung interface motion of these images. Similar to the 4D‐based motion model approach, the correlation model was continuously updated during beam‐on imaging based on the most recent 30 dynamics before use.

### 2.3 Multileaf collimator tracking

#### 2.3.1 MR imaging

Tracking experiments were performed with the previously described beam‐on SMS sequences. The SMS‐TSE (3.15 Hz) and SMS‐bTFE (2.35 Hz) sequence were continuously acquired covering a stack of 28 slices (2(CC)× 2.5(LR)× 4.5(AP) mm^3^ voxel size) adjusted to the dimensions of the phantom. For comparison, a 4 Hz coronal 2D fast field echo (FFE) sequence (fixed slice location) was used as input for MLC tracking. Further scan parameters of this 2D‐FFE sequence can be found in the Supplementary Materials (Table S1).

#### 2.3.2 Real‐time data streaming

To avoid latency during the SMS reconstruction, we enabled real‐time streaming of the SMS data. To this end, we enabled the instantaneous transmission of the k‐space data to the Philips reconstructor after acquiring each pair of SMS slices. Furthermore, we modified the vendor reconstruction software to start reconstructing each slice as soon as the data were received, instead of buffering the data of all slices. The reconstructed images were then streamed via a low latency streaming interface^32^ to a message broker (RabbitMQ 3.8.9)^33^ and processed as described in Section 2.2.2.

#### 2.3.3 Latency measurements

The latency between target motion and MLC response must be minimized to accurately update the position of the radiation beam based on the target position.^15^, ^34^, ^35^ The latency was determined using the motion phantom that followed a sinusoidal trajectory (A = 20 mm, T = 4 s) while images were acquired. These images were timestamped by a client program and the phantom reported positions (with timestamps) were sent to the same client computer. The minimum latency (τ_min_) in MLC tracking consists of the three components shown in Equation (1):^15^

(1) $$\tau_{\min}=T_{\mathrm{signal}}+T_{\mathrm{proc}}+T_{\mathrm{MLC}}$$

*T*_signal_ is the signal acquisition time, which is the time between sampling the center of k‐space and receiving the image. *T*_signal_ was determined by calculating the phase shift between the positions extracted from the timestamped images and the phantom reported positions.^34^ *T*_proc_ equals the image processing time consisting of target position extraction and calculating the MLC aperture accordingly, while *T*_MLC_ equals the MLC adjustment time.^15^

The average latency (τ_average_) is defined by adding half the slice acquisition time to the minimum latency (Equation 2):

(2) $$\tau_{\mathrm{average}}=\tau_{\min}+\frac{T_{\mathrm{acq}}}{2}$$

With the integrated electronic portal imaging device and setup used by Uijtewaal et al.,^15^ the average latency was estimated by calculating the phase shift between the two extracted sinusoidal trajectories of a square aperture and a spherical target.^36^

#### 2.3.4 Tracking experiments

The Quasar MRI^4*D*^ phantom was programmed with either Lujan motion (cos^4^, A = 20 mm, T = 4 s), or patient‐derived respiratory motion (A = 11 mm, T = 3 s, 0.6 mm/min drift). Only beam‐on imaging was performed during the tracking experiments. A mid‐position reference was derived by imaging the static phantom in its mid‐position location. For the experiments using the SMS sequences, the SMS slice that intersected the cylindrical phantom insert was used to determine the target (3 cm diameter sphere) position relative to the mid‐position reference. With this target position, the MLC aperture was updated every 40 ms based on predicted positions. The system latency was compensated for by using a previously developed linear (ridge) regression predictor that was continuously retrained.^15^ For comparison, a static reference (no motion) and experiments without tracking (both motion trajectories) were performed.

#### 2.3.5 Dosimetry

A 15‐beam 8× 7.5 Gy intensity‐modulated radiotherapy plan was created following the clinical planning template for lung SBRT using isotropic 3 mm gross tumor volume (GTV) to planning target volume (PTV) margins. Gafchromic EBT3 films were used to evaluate the delivered dose. Local gamma analyses with a gamma evaluation criterion of 3%/3, 2%/2, and 1%/1 mm were performed to quantify dose differences between static and tracking deliveries.^37^ Areas of the film receiving >10% of the prescribed dose were analyzed, whereas areas of low dose were excluded because of calibration uncertainties. Dose profiles were extracted from the films to determine overdosage outside the PTV. The percentage overdosage was determined by calculating the ratio between the dose profiles of the tracking experiments and the static reference scenario. Dose area histograms (DAHs) were used to assess the GTV and PTV coverage, and the percentage target coverage (PTC) was determined for the PTV using a volumetric definition of coverage.^38^, ^39^ The PTC compares the intersection of the PTV and prescription iso‐dose volume (PIV) with the PTV (Equation 3):

(3) $$\mathrm{PTC}=\frac{\mathrm{PTV}\cap \mathrm{PIV}}{\mathrm{PTV}}\times 100\%$$

### 2.4 Validation of the hybrid 2D/4D MRI methodology

#### 2.4.1 Real‐time motion

All beam‐on (navigator) images were used retrospectively to obtain a self‐sorting signal as was described in Section 2.2.2. This self‐sorting signal served as plausibility check for the real‐time motion signals extracted with and without updated 4D‐based motion model. For the in‐silico data, the simulated motion traces served as ground‐truth.

#### 2.4.2 Correlation model

To validate the prebeam‐derived correlation model, motion was predicted with a correlation model derived from images acquired with a 4 Hz coronal 2D‐bTFE sequence (200 dynamics). The predicted spleen–lung interface motion using both correlation models was compared to independently determined ground‐truth motion extracted using template matching (normalized cross‐correlation). For the in‐silico data, ground‐truth reference volumes were available for each time point. This allowed the predicted tumor location to be compared with the actual tumor location.

#### 2.4.3 Beam‐on 4D‐MRI reconstruction

Beam‐on mid‐positions were derived from the dynamically derived beam‐on 4D‐MRIs. The liver–lung interface in the sagittal plane of the beam‐on mid‐position images was rigidly registered to the prebeam‐derived mid‐position to detect baseline drifts. The extracted baseline drifts were compared to mid‐position drifts derived from the corresponding real‐time motion signals.

## 3 RESULTS

### 3.1 Hybrid 2D/4D‐MRI methodology validation

#### 3.1.1 Real‐time motion performance

**FIGURE 2:**
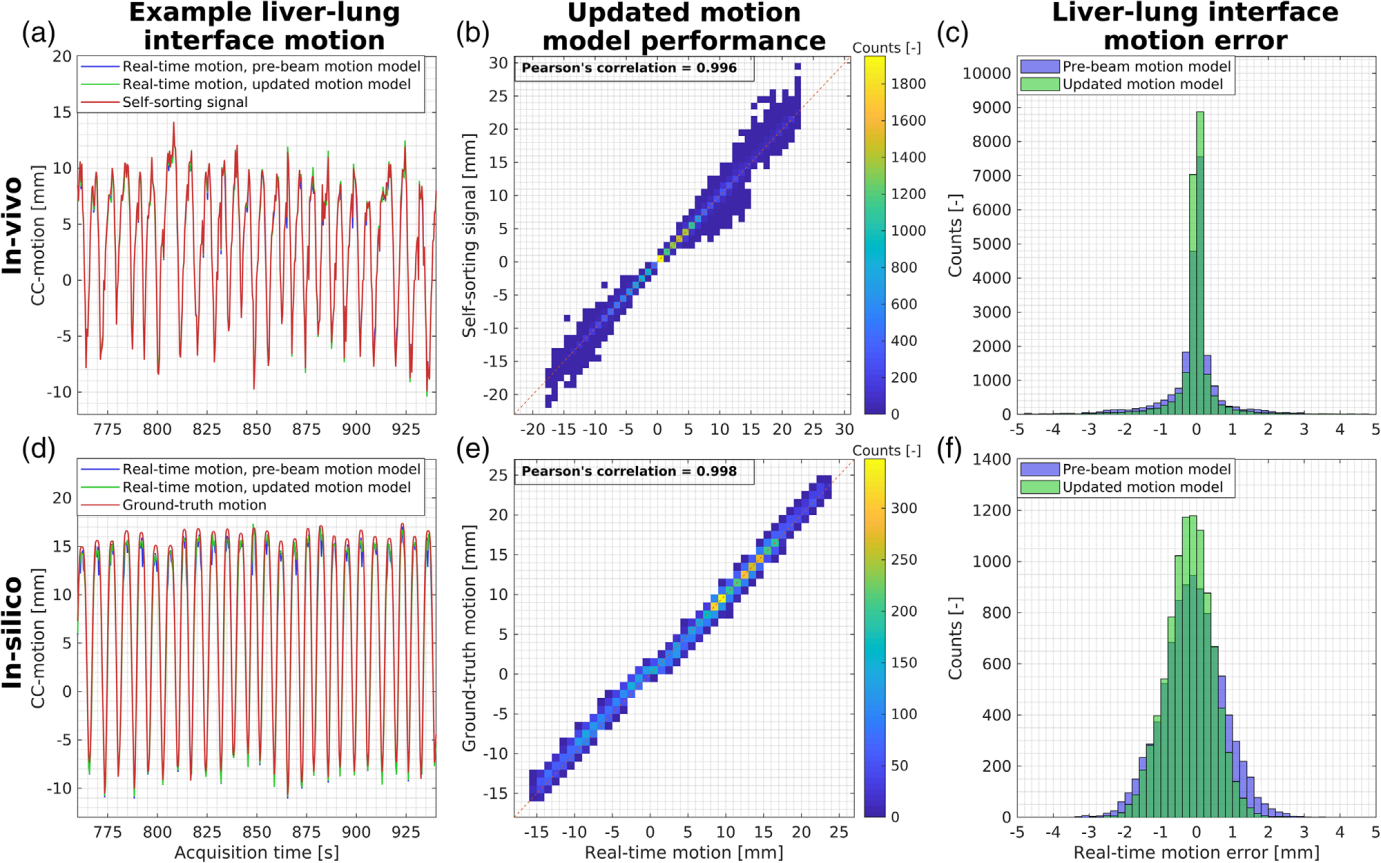
In‐vivo and in‐silico real‐time motion estimation performance. The first column shows examples of real‐time motion estimated with and without updating the prebeam 4D‐based motion model together with reference motion. The second column shows the real‐time motion estimation with the updated 4D‐based motion model versus reference motion. The third column shows the error distribution of the estimated real‐time motion

Overall, an excellent agreement between real‐time motion and the self‐sorting signal was found (Figure 2). Pearson's correlations of 0.996 (in‐vivo) and 0.998 (in‐silico) were found for real‐time motion estimation with updated 4D‐based motion model. Continuously updating the prebeam 4D‐based motion model decreased the median (5th–95th percentile) error of the real‐time motion estimation from 0.0 (−1.8 to 1.0) mm to 0.0 (−0.9 to 0.7) mm averaged over all in‐vivo datasets (Figure 2c). For the in‐silico data, the median (5th–95th percentile) error between real‐time motion and the self‐sorting signal decreased from 0.0 (−0.5 to 1.1) mm to 0.0 (−0.2 to 0.2) mm averaged over the four simulated scenarios. Compared to the ground‐truth motion, the median (5th–95th percentile) error was slightly larger and increased from −0.1 (−1.5 to 1.3) mm to −0.2 (−1.3 to 0.8) mm (Figure 2f). Additionally, analysis of the liver–lung interface motion in the coronal slice intersecting the tumor revealed a median (5th–95th percentile) error of 0.1 (−1.1 to 1.9) mm for the predicted motion compared to ground‐truth motion.

#### 3.1.2 Target motion prediction performance

**FIGURE 3:**
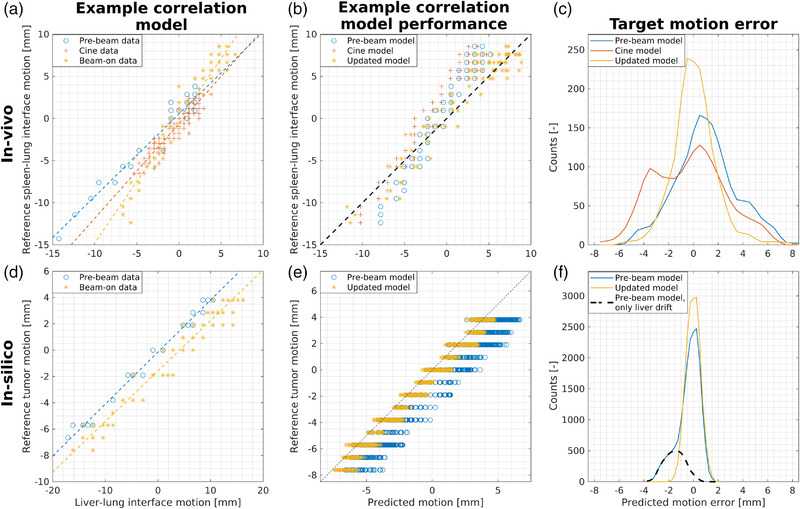
In‐vivo and in‐silico motion prediction performance. The first column shows examples of the relation between liver–lung interface motion and reference motion and the corresponding extracted linear correlation model (dashed lines). The second column shows the corresponding predicted target motion. The third column shows the error of the predicted target motion of all datasets. For in‐vivo, the simulated scenario with only liver drift is depicted as example. Furthermore, it is depicted by the black dashed line in (f), where it represents the tail in the distribution of the prebeam correlation model

Figure 3 shows the prediction performance target motion for in‐vivo (spleen–lung interface) and in‐silico (tumor). Examples are shown where the linear relation between liver–lung interface motion and target motion changed over time. Updating the linear correlation model improves the motion prediction. The spleen–lung interface motion prediction had a median (min–max) root mean square error (RMSE) of 2.3 (1.1–5.1) mm and 2.9 (1.5–3.8) mm, respectively, for the prebeam and cine‐derived correlation models, which was decreased to 1.6 (0.9–2.6) mm when updating the prebeam‐derived correlation model (Figure 3c). Tumor motion prediction had a median (min–max) RMSE of 0.5 (0.5–1.7) mm for the prebeam‐derived model, which decreased to 0.5 (0.5–0.6) mm when updating the prebeam‐derived correlation model. The difference in maximum RMSE was the result of the scenario where the liver drifted, while the tumor did not drift. The black dashed line in Figure 3f shows the contribution of that scenario of the total tumor motion error distribution using the prebeam correlation model.

#### 3.1.3 Beam‐on 4D‐MRI reconstruction

**FIGURE 4:**
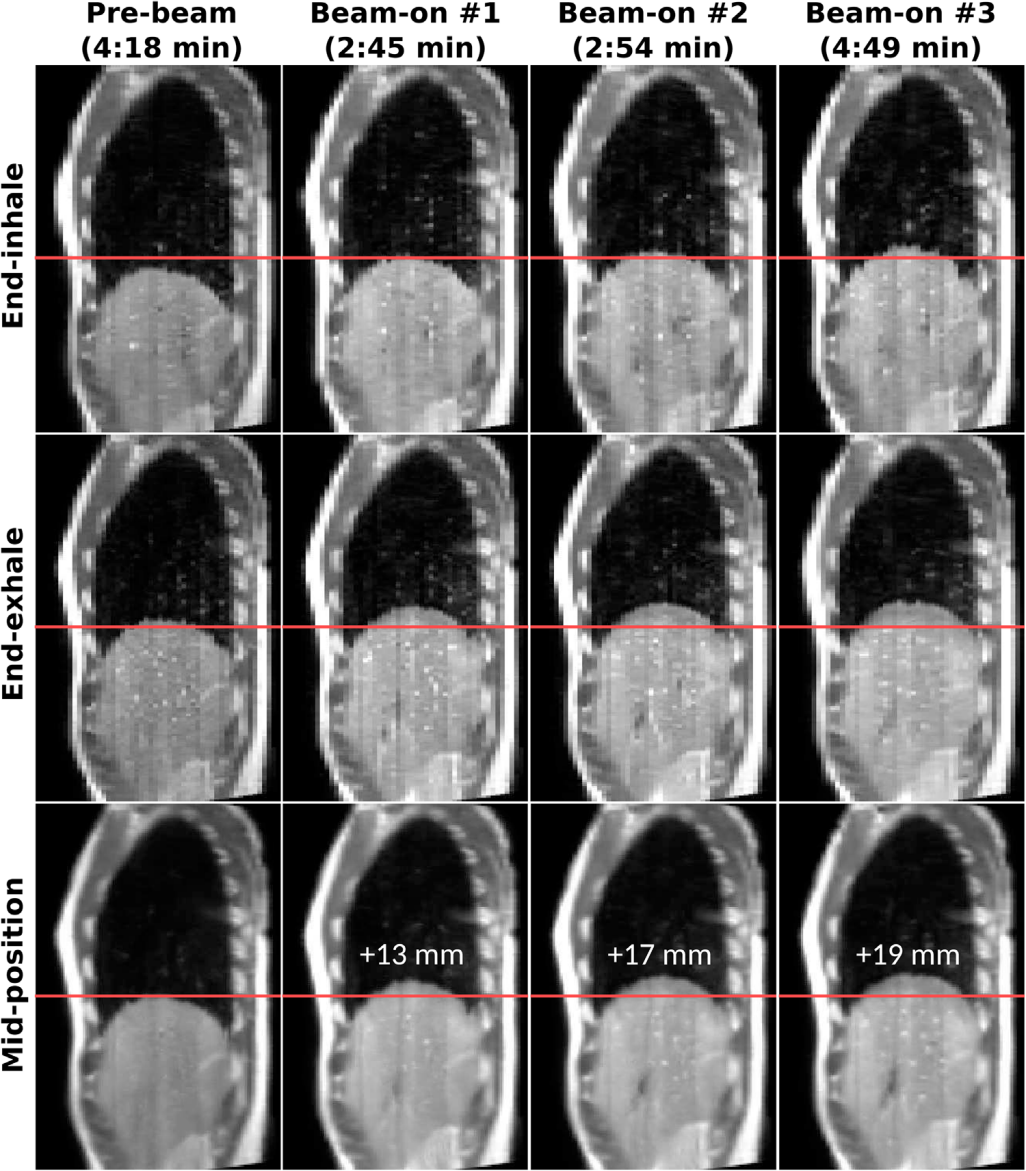
Example volunteer end‐inhale and end‐exhale respiratory phases of prebeam and beam‐on sorted 4D‐MRIs and their derived mid‐position images. Time between brackets denotes the corresponding acquisition time. The horizontal line shows the prebeam mid‐position liver dome location and the numbers represent the amount of cranial drift

The dynamic 4D‐MRI reconstruction required a median acquisition time of 3:18–4:25 min (24 dynamics) to meet the requirement of maximum 10% of missing data. The shortest beam‐on 4D‐MRI took 2:21–3:17 min (17 dynamics) and the longest beam‐on 4D‐MRI took 7:51–10:28 min (57 dynamics). Figure 4 shows an example of sorted 4D‐MRIs (end‐inhale and end‐exhale respiratory phases) and derived mid‐position images for the prebeam and beam‐on phase for the volunteer with the largest amount of drift. For all volunteers, baseline drift of the liver–lung interface extracted from the mid‐position images and their corresponding self‐sorting signals had a median (min–max) difference of 0.2 (−1.7 to 2.3) mm.

### 3.2 Multileaf collimator tracking

#### 3.2.1 Latency

Table 1 summarizes the determined system latencies. For τ_min_, the *T*_proc_ (10 ms) and *T*_MLC_ (92 ms) were adopted from previous work by Uijtewaal et al.^15^ The measured system latency with (without) predictor was −10 ± 4 (398 ± 10) ms, −1 ± 3 (501 ± 12) ms, and 0 ± 3 (319 ± 9) ms for the SMS‐bTFE, SMS‐TSE, and 2D‐FFE sequences, respectively. Although the two SMS sequences have more latency compared to the 2D‐FFE sequence, the predictor was able to compensate this. The small negative latency for the SMS sequences means that the predictor slightly overcompensated for the latency.

**TABLE 1:** System latencies measured for three MRI sequences during multileaf collimator tracking

MRI sequence	T_signal_ (ms) (measured)	τ_min_ (ms) (derived)	τ_average_ (ms) (measured)	$\tau_{\mathrm{average}}^{w/\;\mathrm{predictor}}$ (ms) (measured)
SMS‐bTFE	140 (±3)	242 (±6)	398 (±10)	–10 (±4)
SMS‐TSE	274 (±2)	376 (±5)	501 (±12)	–1 (±3)
2D‐FFE	103 (±1)	205 (±5)	319 (±9)	0 (±3)

*Note*: The standard deviation is denoted between brackets.

Abbreviations: bTFE, balanced turbo field echo; FFE, fast field echo; SMS, simultaneous multislice; TSE, turbo spin echo.

#### 3.2.2 Local gamma analysis

Treatment delivery time was 6.2 min, and a median (min–max) film registration error of 0.4 (0.2–0.5) mm was found. Table 2 summarizes the percentage of pixels that passed the local gamma criteria of 1%/1, 2%/2, and 3%/3 mm for all motion management experiments during two types of motion. Applying tracking increases the gamma pass rates by 24.5–55.2%, depending on the type of motion and evaluation criteria.

**TABLE 2:** Gamma passing rates for pixels receiving >10% prescribed dose comparing image‐guided tracking experiments to a static reference scenario

	Lujan motion			Patient‐derived motion		
Tracking type	1%/1 mm	2%/2 mm	3%/3 mm	1%/1 mm	2%/2 mm	3%/3 mm
No	28.2	51.4	64.0	38.9	63.0	75.2
SMS‐bTFE	83.4	99.6	99.9	92.9	98.4	99.9
SMS‐TSE	96.9	99.7	100.0	91.4	98.6	99.7
2D‐FFE	86.9	96.3	99.3	96.7	99.7	99.9

*Note*: Lujan motion (cos^4^, A = 20 mm, T = 4 s) and patient‐derived respiratory motion (A = 11 mm, T = 3 s, 0.6 mm/min drift) were simulated.

Abbreviations: bTFE, balanced turbo field echo; FFE, fast field echo; SMS, simultaneous multislice; TSE, turbo spin echo.

**FIGURE 5:**
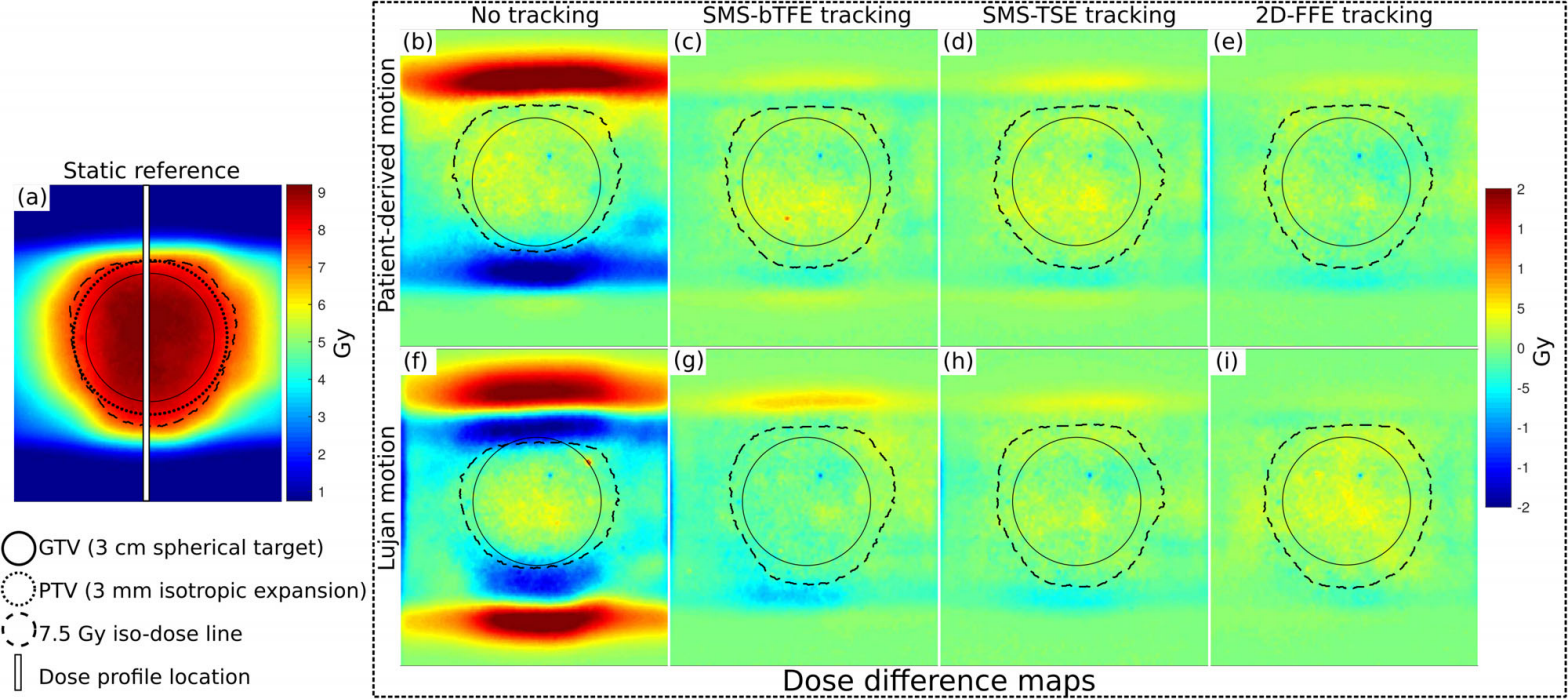
Dosimetric maps of the phantom experiments performed. The dose difference maps compare to the static reference dose map on the left (a). No tracking and tracking using three imaging sequences were performed for patient‐derived motion (b–e) and Lujan motion (f–i)

Figure 5 shows the static reference dosimetric map and dose difference maps compared to the static reference measurement. It shows that the target is underdosed without tracking, and differences of up to 2 Gy (27% of prescription dose) are found compared to the static reference. Furthermore, the figure shows the improvement of conformity when MLC tracking is applied and also shows the excellent agreement between MLC experiments based on SMS and 2D‐FFE imaging. The experiment with SMS‐bTFE imaging and Lujan motion shows some underdosage near the 7.5 Gy iso‐dose line at the caudal side and some overdosage at the cranial side.

#### 3.2.3 Dose profiles

**FIGURE 6:**
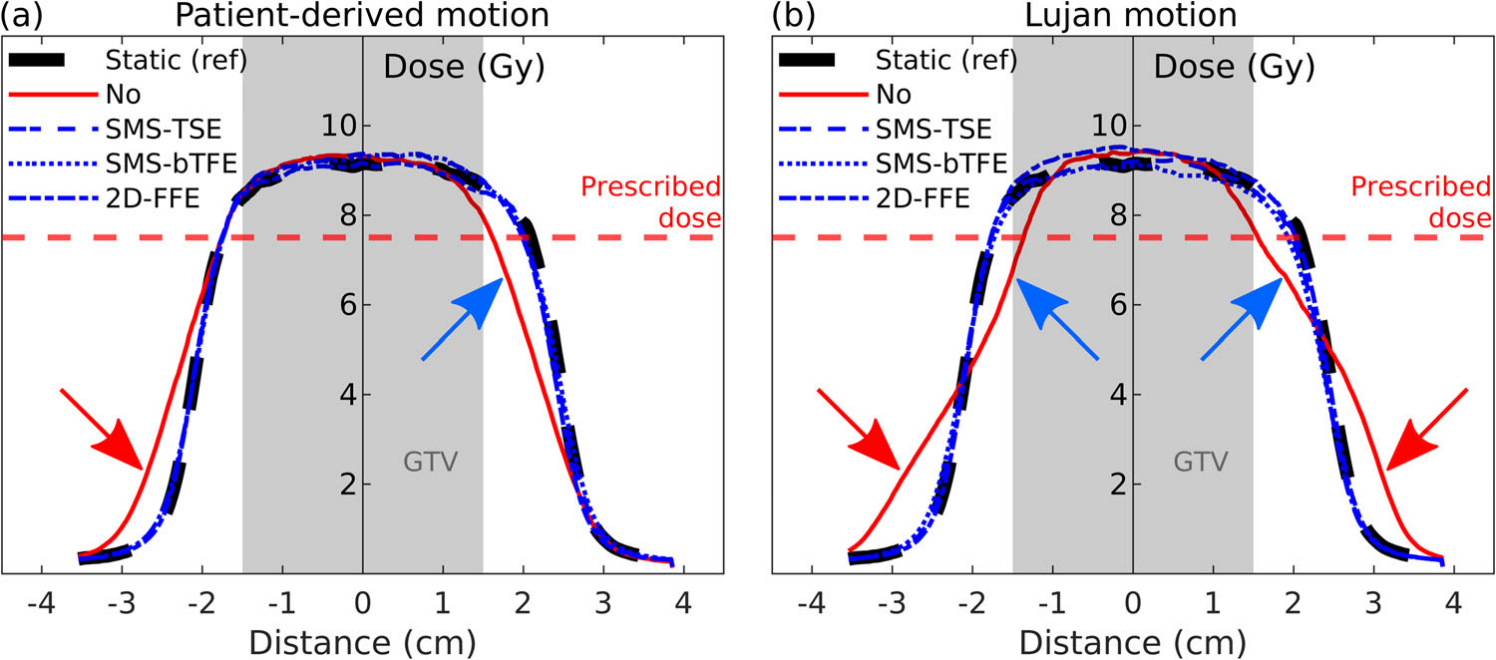
Dose profiles in cranial‐caudal direction for patient‐derived motion (a) and Lujan motion (b). Line types indicate the type of tracking, which were compared to the reference static delivery (black dashed line). Arrows indicate regions of underdosage (blue arrow) and overdosage (red arrow). Note that the blue lines representing the tracking scenarios are superimposed.

Figure 6 shows dose profile comparisons in CC‐direction of the performed tracking experiments. The dose profiles show excellent agreement with the static reference when tracking was applied, while the dose profiles of no tracking deviate and show underdosage and overdosage (arrows Figure 6). The dose profiles of tracking the patient‐derived motion revealed an overdosage (outside the PTV) between 6% and 20% compared to the static reference, which was 203% when no tracking was applied. For Lujan motion, the tracking experiments had an overdosage (outside the PTV) between 5% and 34% compared to the static reference, while for no tracking, an overdosage of 289% was found.

#### 3.2.4 Dose area histograms

The largest differences in GTV coverage are found for the minimum dose ($D_{98\%}$) of the experiments with the Lujan motion compared to a static reference (Table 3). A relative coverage of 97% (SMS‐bTFE) and 100% (SMS‐TSE) was obtained for tracking experiments with the 4D‐MRI sequences, while without tracking, only a relative coverage of 91% was obtained. The latter is depicted in Figure 5, in which the prescribed 7.5 Gy iso‐dose line is inside the spherical target at the cranial side. The median dose ($D_{50\%}$) and maximum dose ($D_{2\%}$) were comparable for all experiments and had a relative coverage between 99% and 103%. For experiments with the patient‐derived motion, all relative GTV coverage values were between 99% and 102%. The relative PTV coverage ($D_{95\%}$) increased by 11–13% for Lujan motion when tracking was applied, while it increased by 3–4% for patient‐derived motion. The PTC of the PTV remained excellent during the tracking experiments that were performed, with coverage values between 98% and 100% for both motion trajectories. When no tracking was applied, the PTC decreased to 87% for Lujan motion and to 97% for patient‐derived motion.

**TABLE 3:** Dosimetric summary for the gross tumor volume (GTV) and planning target volume (PTV), comparing image‐guided tracking experiments to a static reference scenario

		Tracking type			
	Motion type	No	SMSbTFE	SMSTSE	2DFFE
DAH^e^ GTV	No				
	D_98%_ (Gy)	8.28	–	–	–
	D_50%_ (Gy)	8.89	–	–	–
	D_2%_ (Gy)	9.20	–	–	–
	Lujan				
	$\;D_{98\%}^{\mathrm{rel}}$ (%)	91	97	100	100
	$D_{50\%}^{\mathrm{rel}}$ (%)	100	99	100	102
	$\;D_{2\%}^{\mathrm{rel}}$ (%)	102	99	101	102
	Patient‐derived				
	$\;D_{98\%}^{\mathrm{rel}}$ (%)	99	100	101	99
	$D_{50\%}^{\mathrm{rel}}$ (%)	101	101	101	100
	$\;D_{2\%}^{\mathrm{rel}}$ (%)	102	102	102	100
DAH PTV	No				
	D_95%_ (Gy) Lujan	7.89	–	–	–
	$\;D_{95\%}^{\mathrm{rel}}$ (%) Patient‐derived	87	98	100	99
	$D_{95\%}^{\mathrm{rel}}$ (%)	97	100	101	100
PTC^f^ PTV	No				
	Coverage (%) Lujan	100	–	–	–
	Coverage (%) Patient‐derived	87	98	99	99
	Coverage (%)	97	99	100	100

*Note*: No motion, Lujan motion (cos^4^, A = 20 mm, T = 4 s), and patient‐derived respiratory motion (A = 11 mm, T = 3 s, 0.6 mm/min drift) were simulated.

Abbreviations: bTFE, balanced turbo field echo; DAH, dose area histogram; FFE, fast field echo; PTC, percentage target coverage; SMS, simultaneous multislice; TSE, turbo spin echo.

## 4 DISCUSSION

The implemented hybrid 2D/4D‐MRI methodology successfully demonstrated its ability for fast 2D imaging while simultaneously obtaining respiratory‐correlated 4D‐MRIs to support MLC tracking and dose accumulation in abdominothoracic radiotherapy on the Unity MR‐linac. First, the imaging component of the methodology was validated using in‐vivo and in‐silico data. Updating the prebeam‐derived 4D‐based motion model during beam‐on imaging improved the real‐time motion estimation, and a median acquisition time of 2:21–3:17 min was sufficient to dynamically reconstruct contiguous 4D‐MRIs with a maximum of 10% of missing data. In addition, validation of the correlation model showed good prediction of motion beyond the liver dome. Second, analyses of delivered dose during MLC tracking experiments were performed to validate a complete hybrid 2D/4D‐MRI‐based real‐time adaptation pipeline. Dosimetric results were comparable to tracking experiments using a (single‐slice) 2D‐FFE sequence, showing the potential of MLC tracking based on an SMS‐4D‐MRI sequence that simultaneously acquires 4D‐MRI data for dose accumulation.

Two different SMS‐4D‐MRI sequences were used in this study resulting in both T2/T1‐weighted and T2‐weighted images, demonstrating the potential of the hybrid 2D/4D‐MRI methodology for multiple anatomical sites. This is in contrast to the work presented by Mickevicius et al.,^26^ which is limited to gradient spoiled MRI sequences. Moreover, our SMS sequence acquires parallel slices that do not have a saturation band caused by overlapping orthogonal slices. However, a limitation of our balanced SMS‐4D‐MRI sequence (giving T2/T1‐weighted images) is the off‐resonance effects, resulting in bands of low signal. For one dataset, this caused a (local) band of low signal at the liver–lung interface. For the template matching, an additional step to make binary images was necessary to obtain reliable results. Despite this limitation, we were still able to process the data and obtain reliable results. In the future, the off‐resonance effect (band of low signal) around the liver–lung interface could be avoided by changing the off‐resonance location if necessary. Determining the locations of the off‐resonance effect needs a (short) additional scan(s) using the balanced MRI sequence.^40^

To overcome the limitation of not capturing the treatment target with every single slice (as is necessary to achieve volume coverage for the 4D‐MRIs), a 4D‐based motion model was used to obtain estimated unified real‐time liver–lung interface motion of the coronal slice intersecting the treatment target. Updating the 4D‐based motion model decreased the 5th–95th percentile range of real‐time motion error from −1.8 to 1.0 mm and −0.9 to 0.7 mm (in‐vivo) and from −0.5 to 1.1 mm and −0.2 to 0.2 mm (in‐silico) when compared to the self‐sorting signal. Comparing the in‐silico real‐time motion estimation to ground‐truth motion, the median error increased from −0.1 to −0.2 mm, while the 5th–95th percentile range decreased from −1.5 to 1.3 mm and −1.3 to 0.8 mm. Despite the slight higher error when compared to the ground‐truth motion, this shows that real‐time motion can be estimated accurately. In addition, the motion of the liver–lung interface in the slice intersecting the tumor was evaluated for each simulated volumetric dataset resulting in a median (5th–95th percentile) error of 0.1 (−1.1 to 1.9) mm. This is comparable to work by Liu et al.,^41^ which found an average motion error of 0.8 mm for their XCAT validation study using eight phase bins. The SMS data were acquired without (2D) GNL correction to perform offline 3D GNL correction of the 4D‐MRIs.^29^ Future research should investigate if real‐time motion estimation improves if 2D GNL corrected SMS slices are registered to a 2D or 3D GNL corrected mid‐position volume.

The validity of the correlation model is critical to predict tumor motion beyond the liver dome. The in‐vivo data were acquired in healthy volunteers, meaning no tumor was present to build a correlation model. Using an anatomical landmark in the lungs was challenging due to out‐of‐plane motion and signal intensity variation due to blood pulsation. Therefore, the spleen–lung interface motion was used. The nonupdated correlation model had a median RMSE of 2.3 mm and the cine‐derived model had a median RMSE of 2.9 mm. This shows that a prebeam‐derived linear correlation model using only 30 data points that spans a time duration of approximately 5 min can correctly correlate target motion with liver dome motion. Updating the correlation model improved the median RMSE to 1.6 mm, demonstrating that the linear correlation model is susceptible to nonlinear drift of the target and the liver–lung interface and, therefore, should be updated. This was confirmed by the in‐silico study performed, where the maximum RMSE decreased from 1.7 to 0.6 mm, which represented the simulated scenario with only liver drift and no tumor drift.

To take baseline drift into account for dose accumulation, the acquired SMS slices are sorted into respiratory correlated 4D‐MRIs during the beam‐on imaging phase. A maximum of 10% of missing data in sorted 4D‐MRIs was set as inclusion criterion, which was deemed acceptable as the mid‐position generation can compensate for missing data in certain phases by augmenting with data acquired in other phases.^31^ With this inclusion criterion, beam‐on 4D‐MRIs were reconstructed requiring a median of 24 dynamics, which is 20% less than the currently used number of dynamics during prebeam imaging. One beam‐on 4D‐MRI required 57 (+90%) dynamics because that volunteer was taking deep breaths at the beginning of the acquisition and shallower breaths later on. These deep breaths gave a large peak‐to‐peak amplitude between the end‐inhale and end‐exhale bin, which led to missing data in the respiratory phases near end‐inhale when switched to shallow breathing. The longer the shallow breathing continued, the smaller the peak‐to‐peak amplitude of the bins became due to the dynamically changing bin edges. This resulted in less missing data in the respiratory phases near end‐inhale. A regular breathing pattern might yield better sorting performance. A pragmatic solution could be to coach volunteers or patients upfront, or similarly provide feedback on their breathing pattern through an in‐room screen to regularize breathing.^42^, ^43^ Extracted baseline drifts from the derived mid‐position volumes agreed within 2.3 mm with the corresponding self‐sorting signals, which decreases to 1.7 mm if the previously discussed 57‐dynamics beam‐on 4D‐MRI is not taken into account. This is smaller than the acquisition voxel size of 2 mm, conforming the robustness of the method of detecting baseline drift.

This effect of nonlinear drift between target motion and liver dome motion was confirmed by the in‐silico validation. The median error improved from −0.2 to −0.0 mm when the correlation model was updated, mainly as a result of the simulated scenario where only the liver drifted. A limitation of the in‐silico data was the relatively regular motion traces that were simulated. Although some variation in breathing amplitude and frequency was implemented, it did not represent irregular breathing that can be found in‐vivo. Another limitation was the simplicity of intensity values in the XCAT phantom used, which did not contain noise. However, the analysis of the in‐silico data still showed that the implemented methodology can accurately estimate tumor motion beyond the liver dome, based on liver–lung interface motion in combination with the 4D‐based motion model and correlation model.

Dosimetric analyses were performed to ensure correctness in the extracted target position for MLC tracking. Compared to the reference 4 Hz 2D‐FFE sequence, the SMS‐TSE (3.15 Hz) and SMS‐bTFE (2.35 Hz) MRI sequences were relatively slow. As a result, predictions of 318 and 634 ms (SMS‐TSE), and 425 and 850 ms (SMS‐bTFE) ahead were made by the prediction filter.^15^ Larger look‐ahead times result in larger prediction errors.^44^ This probably explains the area outside the PTV of slight overdosage (34%) seen in Figure 5 for the SMS‐bTFE experiment in combination with Lujan motion, which was 289% when no tracking was applied. Future research should investigate what caused this overdosage outside the PTV for the SMS‐bTFE sequence to improve MLC tracking performance. Despite the high latency, the prediction filter was able to reduce the latency to almost zero, resulting in comparable dosimetric results to 2D‐FFE tracking. A limitation of our MLC tracking experiments is that we focused only on CC‐motion, while the abdominothoracic tumors also move, to a lesser extent, in AP and LR directions.^1^, ^44^ Further research should investigate MLC tracking performance based on correlating CC liver–lung interface motion to 3D tumor motion. An alternative to the multislice MRI sequence could be to use a 3D MRI sequence. This is, however, still challenging due to the need for a subsecond temporal resolution. Recently, promising methods utilizing a 3D acquisition have been developed to estimate 3D motion from minimal k‐space data itself^45^ or from undersampled MR images using a convolutional neural network.^46^

To quantify the dosimetric correctness of the delivered dose, the target coverage was determined. For the tracking experiments with patient‐derived motion, we found a relative GTV coverage between 99% and 102% for all tracking experiments even with no tracking applied. For the PTV, we found a relative coverage of 100% or 101% when tracking was applied, which was reduced to 97% when no tracking was applied. The patient‐derived motion trace had a peak‐to‐peak amplitude of 11 mm with a drift of 0.6 mm/min. This drift, however, does not start until about 8 min after the start of the trace, while the treatment delivery took 6.2 min. Moreover, the spherical target was 3 cm in diameter, and the PTV was a 3 mm isotropic extension of this. The small peak‐to‐peak motion and drift resulted in adequate PTV coverage ($D_{95\%}$ = 7.64 Gy) even without tracking. However, the dosimetric map (Figure 5b) and dose profile (6a) revealed an area (outside target area) of overdosage (203%) that was resolved when tracking was applied (6–20%). Despite the comparable MLC tracking performance of the SMS sequences compared to conventional 2D‐FFE tracking, future experiments should investigate the performance during a patient‐derived motion trace with a larger peak‐to‐peak amplitude and also a fast breathing signal to investigate the sufficiency of the imaging frequency.

## 5 CONCLUSIONS

A novel 2D/4D MRI methodology was developed using an SMS‐accelerated MRI sequence that continuously acquires 2D images and repeatedly cycles through slice positions over the 3D image volume. The individual 2D acquisitions allow for MLC tracking, while the full FOV coverage facilitates the reconstruction of contiguous respiratory‐correlated 4D‐MRIs with a guaranteed percentage of missing data for dose accumulation. Validation of the extracted motion from the 2D images demonstrated the potential for accurately extracting real‐time liver–lung interface motion, while the dosimetric analysis showed comparable results to 2D cine‐MRI tracking despite the longer latency of the SMS sequences. This makes the developed 2D/4D‐MRI methodology a suitable candidate for abdominothoracic radiotherapy guidance.

## CONFLICTS OF INTEREST

The authors have no relevant conflicts of interest to disclose.

## Supporting information

Supporting Information

Supporting Information
