# Supplementary material for: A hybrid 2D/4D‐MRI methodology using simultaneous multislice imaging for radiotherapy guidance
Source: Med Phys. 2022 Jun 22;49(9):6068–81. doi: 10.1002/mp.15802 (PMC9545880; doi:10.1002/mp.15802)
Supplement: Supplementary file 2 — Supporting Information [file MP-49-6068-s002.pdf]

A hybrid 2D/4D-MRI methodology using simultaneous multi-slice imaging for radiotherapy guidance

Katrinus Keijnemans[1], Pim T.S. Borman[1], Prescilla Uijtewaal[1], Peter L. Woodhead[1,2], Bas W. Raaymakers[1], Martin F. Fast[1]
[1]Department of Radiotherapy, University Medical Center Utrecht, Heidelberglaan 100, 3584 CX Utrecht, The Netherlands
[2]Elekta AB, Kungstensgatan 18, 113 57 Stockholm, Sweden

Table S-1: Scan parameters used for the SMS-4D-MRI and 2D cine-MRI scans that were acquired in volunteers on the 1.5 T Unity MR-linac.

**Scan parameter**	**SMS-4D-bTFE**		**SMS-4D-TSE**		**2D-cine in-vivo validation**	**2D-cine tracking experiments**
Sequence type	bTFE		TSE		bTFE	FFE
FOV (CC×LR×AP) [mm^3]	350×457×208–260		350×457×208–260		350×457×10	350×350×10
SENSE	2.5		1.5		1.5	1.5
SMS	2		2		NA	NA
Partial Fourier	0.625		0.625		0.625	0.625
Flip angle [°]	45		110		50	6
Slice orientation	Coronal		Coronal		Coronal	Coronal
Slice acquisition order	Interleaved		Interleaved		Default	Default
REST slabs	2 perpendicular		2 perpendicular		0	0
Geometry correction	No		No		No	2D
Number of slices	52		52		1	1
	Pre-beam	Beam-on	Pre-beam	Beam-on		
Voxel size (CC×LR×AP) [mm^3]	2×2×4–5	2×2.5×4–5	2×2×4–5	2×2.5×4–5	2×2.5×10	2.5×2.5×10
TR/TE [ms]	4.1/2.1	5.0/2.5	8612/58	8266/54	3.6/1.8	4.0/2.0
Shot-length [ms]	397	385	319	284	274	236
Slice acquisition time [ms]	429	424	331	318	277	252
Number of dynamics	30	90	30	90	200	NA
Dynamic scan time [s]	11.2	11.0	8.6	8.3	0.277	0.252
Total scan time [min:s]	5:35	16:32	4:18	12:24	00:55	NA
Total acquired images	1560	4680	1560	4680	200	NA
SAR level [W/kg]	0.9	0.7	2.3	2.0	1.3	0.0
dB/dt [T/s]]	33	15	31	15	30	19
